# Reasons That Lead People to End Up Buying Fake Medicines on the Internet: Qualitative Interview Study

**DOI:** 10.2196/42887

**Published:** 2023-02-16

**Authors:** Hamzeh Almomani, Nilesh Patel, Parastou Donyai

**Affiliations:** 1 Department of Pharmacy Practice Reading School of Pharmacy University of Reading Reading United Kingdom; 2 Department of Pharmacy and Forensic Science School of Cancer & Pharmaceutical Sciences King’s College London London United Kingdom

**Keywords:** fake medicines, prescription-only medicines, internet, theory of planned behavior, interviews, thematic analysis, the United Kingdom

## Abstract

**Background:**

Many people in the United Kingdom are turning to the internet to obtain prescription-only medicines (POMs). This introduces substantial concerns for patient safety, particularly owing to the risk of buying fake medicines. To help reduce the risks to patient safety, it is important to understand why people buy POMs on the web in the first place.

**Objective:**

This study aimed to identify why people in the United Kingdom purchase medicines, specifically POMs, from the internet, and their perceptions of risks posed by the availability of fake medicines on the web.

**Methods:**

Semistructured interviews were conducted with adults from the United Kingdom who had previously purchased medicines on the web. Purposive sampling was adopted using various methods to achieve diversity in participants’ experiences and demographics. The recruitment was continued until data saturation was reached. Thematic analysis was employed, with the theory of planned behavior acting as a framework to develop the coding of themes.

**Results:**

A total of 20 participants were interviewed. Participants had bought various types of POMs or medicines with the potential to be misused or that required a higher level of medical oversight (eg, antibiotics and controlled medicines). Participants demonstrated awareness of the presence and the risks of fake medicines available on the internet. The factors that influence participants’ decision to buy medicines on the web were grouped into themes, including the advantages (avoiding long waiting times, bypassing gatekeepers, availability of medicines, lower costs, convenient process, and privacy), disadvantages (medicine safety concerns, medicine quality concerns, higher costs, web-based payment risks, lack of accountability, and engaging in an illegal behavior) of purchasing medicines on the web, social influencing factors (interactions with health care providers, other consumers’ reviews and experiences, word of mouth by friends, and influencers’ endorsement), barriers (general barriers and website-specific barriers) and facilitators (facilitators offered by the illegal sellers of medicines, facilitators offered by internet platforms, COVID-19 outbreak as a facilitating condition, and participants’ personality) of the purchase, and factors that lead people to trust the web-based sellers of medicines (website features, product appearance, and past experience).

**Conclusions:**

In-depth insights into what drives people in the United Kingdom to buy medicines on the web could enable the development of effective and evidence-based public awareness campaigns that warn consumers about the risks of buying fake medicines from the internet. The findings enable researchers to design interventions to minimize the purchasing of POMs on the web. A limitation of this study is that although the interviews were in-depth and data saturation was reached, the findings may not be generalizable, as this was a qualitative study. However, the theory of planned behavior, which informed the analysis, has well-established guidelines for developing a questionnaire for a future quantitative study.

## Introduction

### Background

Many people use the internet to obtain their medicines, a practice that is attributed to the advantages offered by internet pharmacies, such as convenience, low cost, and round-the-clock accessibility [1]. Legitimate internet pharmacies can supply both medicines that do not require a prescription (called *over-the-counter medicines*) and stronger medicines that require a prescription from an authorized prescriber before they can be legally supplied. In the United Kingdom, the latter are referred to as prescription-only medicines (POMs), a term used hereon for these medicines. Accordingly, consumers in the United Kingdom cannot legally obtain POMs on the web without a prescription from a registered prescriber. However, some people may try to buy POMs without a prescription being issued, and indeed some suppliers may offer POMs via illegal means.

This is because both legal and illegal web-based sellers of medicines operate on the internet. In the United Kingdom, any website selling medicines must be registered with the General Pharmaceutical Council and the Medicines and Healthcare products Regulatory Agency before it can be considered a legal internet pharmacy. Any internet pharmacy that operates outside regulatory systems such as these is deemed illegal. It has been estimated that 96% of all global internet pharmacies are operating illegally [2]. There are serious patient safety concerns around illegal web-based sellers of medicines, who enable a wide range of medicines, including high-risk controlled medicines, to be obtained without a prescription and without medical supervision [3]. Several researchers have explored the prevalence of illegal internet pharmacies and the variety of the products they offer. For instance, UK researchers [4] found that modafinil and methylphenidate (both classified as drugs of abuse with potential for addiction) were available on the web and accessible to UK-based consumers without the need for a prescription. Similarly, another study conducted in the United Kingdom explored the web-based availability of antibiotics, finding that 45% (9/20) of the websites offered antibiotics without the need for a prescription [5].

Purchasing medicines from illegal sellers is associated with many risks, especially if the medicines bought are POMs or require a higher level of medical oversight during the purchase and subsequent use. The risks include the possibility of misuse or abuse of medicines and purchasing contraindicated medicines, which are medicines that should not be used by specific people [6]. Moreover, consuming antibiotics bought on the web without medical oversight could increase antimicrobial resistance [5]. An additional serious problem associated with buying medicines on the web is the risk of buying fake medicines [7]. According to an estimation by the World Health Organization, half of the medicines sold on the web are fake [8]. For example, a study conducted in Japan found that of the 45 samples of tadalafil purchased and tested, 23 (51%) were fake, only 9 (20%) were genuine, and the remaining 13 (29%) were either unregistered medicines or unconfirmed because of insufficient information [9]. Similarly, UAE researchers [10] who bought furosemide tablets on the web and analyzed their physical and chemical properties according to the British Pharmacopoeia (2018) found that they failed to pass the chemical assay test, which means that they were likely fake. In the United Kingdom, millions of fake medicines have been seized at the borders and thousands of websites that offered fake medicines have been shut down by Interpol through operation Pangea in the past few years [11]. Among the medicines seized were antidepressants, erectile dysfunction tablets, painkillers, and slimming pills. Nonetheless, the problem persists.

Several awareness campaigns have been conducted in the past by different civil societies and governmental, and international organizations to warn consumers about the dangers of fake medicines available on the web [12]. In the United Kingdom, the Medicines and Health care products Regulatory Agency has run awareness campaigns on different social media platforms (eg, #FAKEMEDS) that aim to encourage consumers who purchase medicines from the internet to ensure that they are purchasing from legal sources. Despite these efforts, people in the United Kingdom still end up buying medicines from illegal web-based sources. According to an estimate, 10% (1/10) of people in the United Kingdom bought a fake medical product from an illegal web-based source in 2020 [13].

Most of the awareness campaigns have been focused on warning consumers about the risks and dangers of buying POMs on the web as well as educating people about how to safely purchase medicines on the web. However, little attention has been paid to examining the reasons that lead people to buy medicines on the web, with only a limited number of studies exploring this angle [1,14-18]. None of the existing qualitative studies explain why people in the United Kingdom buy POMs on the web without involving their physicians. However, qualitative studies help the development of awareness campaigns that are relevant and fit for purpose by providing insights into why people behave in specific ways.

### Goal of This Study

This study aimed to provide an understanding of why people in the United Kingdom purchase medicines directly from the internet. The focus was on POMs and medicines that have the potential to be misused or that require a higher level of medical oversight during the purchase. This includes some categories of medicines that should not be sold on the web without consultation with a prescriber or without adequate checks made when a prescriber is involved to ensure that they are clinically appropriate, for example, antimicrobials; opioids; sedatives; laxatives; gabapentin; lithium; warfarin; and medicines for diabetes, asthma, epilepsy, and mental health [19]. In addition, this study aimed to explore UK consumers’ perceptions of the web-based availability and risks of fake medicines. UK consumers who had actual experiences of purchasing medicines on the web were interviewed, and the theory of planned behavior (TPB) was used to interpret their behavior.

## Methods

### Overview

This interview study followed the Standards for Reporting Qualitative Research guidelines for the conduct of the research (Multimedia Appendix 1) [20]. Three researchers (HM, PD, and NP) met weekly to manage the project. HM is a PhD student and a novice qualitative researcher who undertook training courses in interviewing structures and techniques and qualitative data analysis. PD is an experienced qualitative researcher and a professor of pharmacy practice. NP is an experienced qualitative researcher and an associate professor of pharmacy practice.

### The TPB

To explore why people purchase medicines directly from the internet (ie, without involving a professional health care provider), the TPB was used as the underpinning theoretical framework. The TPB is one of the psychological theories introduced by Ajzen [21]. It was used in this research to interpret consumers’ behaviors based on their intentions. In this case, the behavior of interest was “purchasing POMs or those medicines that have potential to be misused or that require a higher level of medical oversight during the purchase, through the internet and without involving the doctor.”

**Figure 1:**
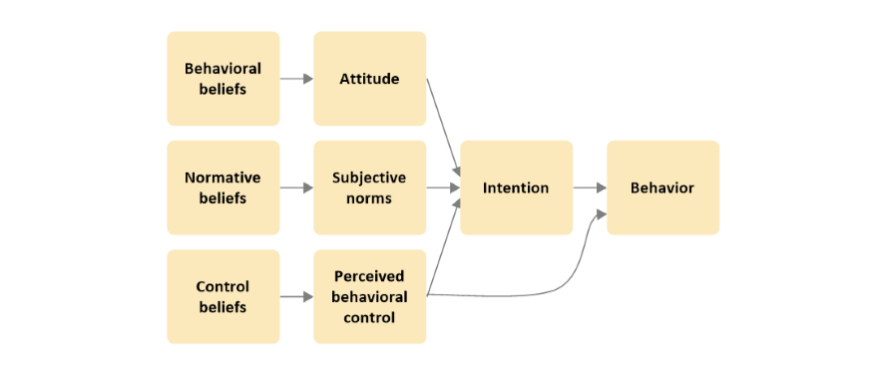
The theory of planned behavior framework.

According to the TPB (Figure 1), consumers’ actual behavior is predicted by their behavioral intentions, which, in turn, are determined by their attitude toward the behavior, subjective norms, and perceived behavioral control [21]. Specific kinds of beliefs underlie each of these 3 predictors of intention: behavioral beliefs, normative beliefs, and control beliefs. These beliefs are the indirect predictors of intention. Behavioral beliefs capture consumers’ beliefs about the outcomes of the behavior, including the perceived advantages and disadvantages of the behavior, and determine the overall attitude toward this behavior. Normative beliefs are consumers’ beliefs about how other specific groups of people would like them to act in relation to the behavior in question or the social pressure and determine subjective norms, which are the individual’s overall perceived expectations from others. Control beliefs are consumers’ specific beliefs about their ability to control the behavior, for example, through access to the resources and opportunities required to facilitate the behavior and determine the individual’s overall perceived behavioral control over the behavior. The logic behind using the TPB in this study is that it has well-established guidelines to enable the development of a questionnaire on the topic in a future study, allowing the factors identified in a qualitative study such as this to be formally verified and generalized at a later date [22]. This investigation would identify the specific elements that are most crucial in driving people’s intentions to buy POMs or other high-risk medicines on the web so that future public awareness campaigns can be evidence based and arguably more effective.

### Trust and the TPB

Trust is an important element influencing consumer behavior [23] and has been proven to be relevant in uncertain environments, such as the context of e-commerce [24]. Trust is a crucial predictor of consumers’ web-based shopping intention and behavior [24,25]. Therefore, consumers’ trust in internet pharmacies can arguably play an important role in influencing their decision to purchase POMs from the internet. In this study, consumers’ trust in web-based sellers of medicines is defined as consumers’ conviction that the web-based sellers will behave according to their expectations by properly delivering effective and safe medicines [26,27].

**Figure 2:**
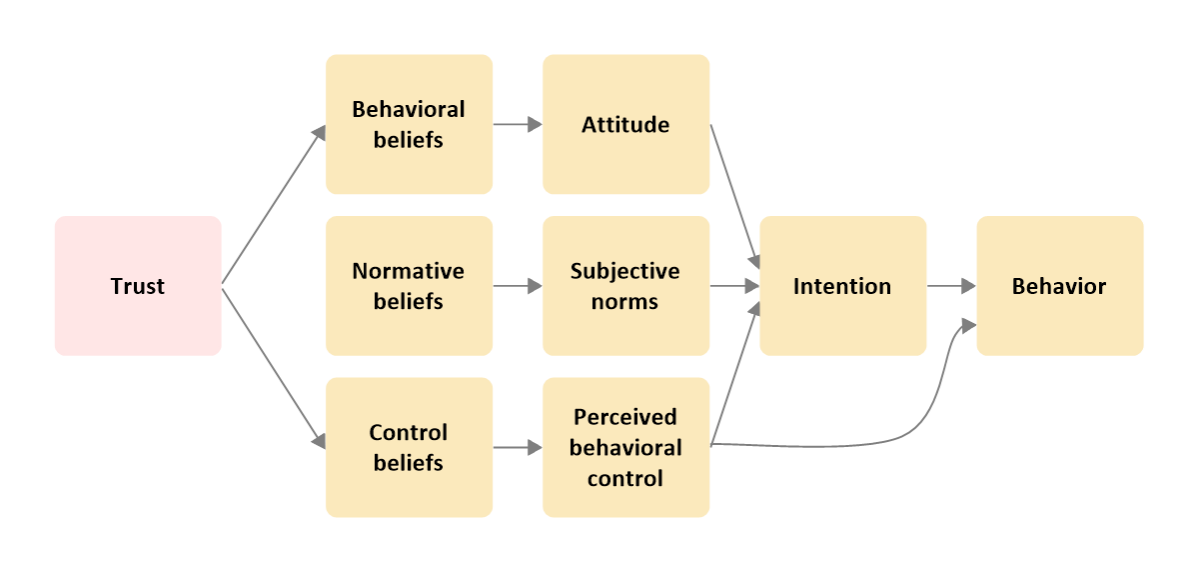
The theory of planned behavior extended model with the addition of trust.

Several researchers have modeled trust in web-based sellers to the TPB framework to explore various behaviors in e-commerce [16,24,28-30]. In this study, an extended model of the TPB framework proposed by Pavlou [24] was adopted by adding consumers’ trust in web-based sellers of medicines as an indirect predictor of consumers’ intentions and a direct predictor of both their attitude (as a behavioral belief) and perceived behavioral control (as a control belief). Trust is proposed as a behavioral belief because it enables consumers’ positive expectation that no harmful outcomes will happen to them, thus creating a favorable perception of the outcomes and, in turn, a positive attitude toward the web-based sellers of medicines [26]. By contrast, trust is also proposed as a control belief, as it builds consumers’ confidence to depend on the web-based sellers of medicines, which helps consumers overcome psychological barriers to engaging in the behavior. In other words, trust serves to absorb uncertainty and facilitate the behavior [24]. Figure 2 illustrates the adopted model.

### Sampling and Recruitment Strategy

The study population was adults (aged ≥18 years) based in the United Kingdom who had experience of purchasing medicines on the web. Additional inclusion criteria were that the participants must be native English speakers and able to access Microsoft Teams (Microsoft Corp) because the interviews were to take place on Teams. The focus was on POMs or medicines that have a potential to be misused or require a higher level of medical oversight during the purchase and subsequent use. Purposive sampling was adopted to achieve diversity in participants’ experiences and demography. Individuals who had purchased POMs before 2019 were excluded to ensure that the participants’ recall of their experiences would not be hindered by a large time gap.

Regarding the recruitment process, the participants were recruited using 3 different procedures. First, emails that included a recruitment poster were circulated to the university staff and students. Second, the same recruitment poster was posted on different social media platforms, including Twitter, Reddit, Facebook, and Telegram. The recruitment poster is presented in Multimedia Appendix 2. Third, a market research company (Panelbase) was employed to assist and boost the recruitment process. Panelbase has >350,000 UK-based adults on their database who agree to being contacted for recruitment to different types of research [31]. The participants were recruited using this company in 3 phases. First, the prevalence of people who had purchased POMs on the web without input from a health care professional was checked by a mini poll sent to 10,473 UK-based adults registered with Panelbase. This mini poll consisted of a single question (ie, *Have you ever bought prescription medicines without involving the doctor?*). The purpose of sending this mini poll was to check the availability of eligible participants in advance via the Panelbase platform. Second, a web-based recruitment screener was developed and sent to those registered with Panelbase (Multimedia Appendix 3). This was a prerecruitment questionnaire used to determine respondents’ eligibility for the study based on the inclusion criteria. Noneligible respondents were excluded from the study at this stage. Moreover, the web-based recruitment screener included demographic questions. In the third phase, emails containing detailed information about the study and consent form were sent to eligible participants with a schedule of potential interview dates. Consent forms were signed by the participants and obtained at least 24 hours before the interviews.

The recruitment process began in April 2021 and ended in May 2022. Regarding the sample size for an interview study, Brinkmann and Kvale [32] recommend recruiting between 5 and 25 participants depending on the study purpose, whereas Kuzel [33] recommended a sample size of 12 to 20 participants; therefore, we set an initial desired sample size between 15 and 25 participants. Recruitment was continued until data saturation was reached, that is, when no more new codes were identified [34]. Each participant was reimbursed £20 (US $24.14) as a token of appreciation for their contribution to the study.

### Data Collection

Semistructured interviews were conducted on the web using the Microsoft Teams app by an interviewer (HM). A semistructured interview schedule comprising open-ended questions to explore the participants’ experience of purchasing medicines on the web was developed (Multimedia Appendix 4). This was developed by all the 3 researchers (HM, PD, and NP) based on the constructs of the model shown in Figure 2. The questions addressed the advantages and benefits of the purchase, the disadvantages and risks involved in the purchase, the facilitators of and barriers to the purchase, the social factors affecting decisions, and why they trusted the web-based supplier. Furthermore, some questions were asked to check the participants’ perception of the web-based availability of and risks associated with fake medicines. A pilot interview was conducted by HM with a PhD student from the University of Reading to pretest the interview schedule and identify questions that were unclear or too complex to understand.

The interviews were video and audio recorded after written consent was received, and consent was verified again verbally at the beginning of each interview.

### Analysis

Interview recordings were transcribed verbatim into an MS Word document by HM. Each transcript was assigned a unique code number for identification (*P-1, P-2, P-3...P-20*), rather than using any other identifying data (eg, names or emails). Any personal information or any research data obtained before, during, or after the interviews were kept confidential on a password-protected computer using a OneDrive (Microsoft Corp) account of HM.

A thematic analysis was performed according to Langdridge and Hagger [35]. Then, the identified themes were categorized against the constructs of the framework shown in Figure 2. The transcripts were read and reread a minimum of 3 times, and line-by-line notes were made. The interview transcripts were analyzed iteratively using the NVivo (version 12; QSR International) software to code and organize the data. Three coding levels were used. First order coding was the first level of coding, which was descriptive with minimal interpretation of data. Then, second order coding was conducted by linking related first-order codes to create the themes. The analysis was repeated for each transcript, and a summary document was produced for each participant. The summaries of themes for all the participants were subjected to third order coding, whereby higher-level themes (ie, superordinate themes) were identified. Then, the superordinate themes were categorized against the constructs of the framework shown in Figure 2. The coding process and analysis were carried out by HM and reviewed step by step by PD and NP.

### Ethics Approval

Ethics approval was obtained from the ethics committee of University of Reading (reference number 21_07).

## Results

### Participants’ Characteristics

A total of 20 participants (n=12, 60% females, and n=8, 40% males) were recruited and interviewed. Of these 20 participants, 1 (5%) was recruited through the emails sent to the university staff and students, 4 (20%) were recruited using social media platforms, and 15 (75%) were recruited using Panelbase. Overall, 85% (17/20) of participants were White British, 10% (2/20) of participants were Asian British, and 5% (1/20) of participants were Black British. The participant age groups were 18 to 29 (4/20, 20%), 30 to 39 (4/20, 20%), 40 to 49 (5/20, 25%), 50 to 59 (4/20, 20%), and ≥70 (3/20, 15%) years. In total, 60% (12/20) of participants were from England, 30% (6/20) were from Scotland, and 10% (2/20) were from Northern Ireland. The interviews lasted from 34 to 75 (average duration 48, SD 9.39) minutes.

### Participants’ Experiences

To the mini poll of the 10,473 UK-based adults registered with Panelbase, 1321 (12.61%) responded, of whom 136 (10.3%) indicated they had bought POMs on the web without involving their physician. This reassured the team that eligible participants might be identified during recruitment.

The 20 participants interviewed had bought various types of POMs or medicines with the potential to be misused or that required a higher level of medical oversight (eg, hormone replacement therapy, antibiotics, and high-risk controlled medicines). Table 1 shows each participant’s purchasing experiences. All the participants were the end users of the medicines purchased, except for participant 9 (P-9), who had bought medicines on the web for both himself and his mother.

Most of the participants did not report encountering problems with the medicines purchased or with the suppliers of these medicines. However, a limited number of participants (3/20, 15% participants) did report encountering problems, including failure in delivery, financial losses, and product quality issues, as stated in the following quotes:

I’ll give you an example for my case. Uhm, I ordered from a website in Russia which was shut down under a basic like they’re rounding up online websites there, so I lost money on about six months’ worth of HRT. You know, that was a pretty devastating for me.

P-2, line 194

I bought Tramadol once and they sent me these tiny little orange pills instead that.

P-5, line 124

I found that the Valium felt stronger than the ones from the doctors.

P-19, line 134

**Table 1:** Participants’ experiences.

Code	Number of purchases	Last purchase date (interview date)	Problems with the purchase	Medicines bought on the web
P-1	≥3	Not provided (2021)	No	Names were not provided
P-2	≥3	2021 (2021)	Yes	Estradiol and spironolactone
P-3	≥3	2021 (2021)	No	Estradiol and spironolactone
P-4	≥3	2021 (2021)	No	Estradiol and spironolactone
P-5	≥3	2021 (2021)	Yes	Codeine and tramadol
P-6	≥3	2022 (2022)	No	Senna (100 tablets)
P-7	1	2020 (2022)	No	Acetazolamide and amitriptyline
P-8	≥3	2020 (2022)	No	Sildenafil
P-9	2	2022 (2022)	No	Paroxetine, brimonidine ED^a^, and timolol ED
P-10	2	2022 (2022)	No	Clonazepam and sildenafil
P-11	2	2022 (2022)	No	Letrozole
P-12	≥3	2019 (2022)	No	Varenicline
P-13	1	2020 (2022)	No	Tranexamic acid
P-14	2	2021 (2022)	No	Lymecycline and metronidazole
P-15	≥3	2021 (2022)	No	Sildenafil
P-16	2	2021 (2022)	No	Norethisterone
P-17	≥3	2021 (2022)	No	Hydrocodone-paracetamol, alosetron, and antibiotic
P-18	≥3	2021 (2022)	No	Sumatriptan
P-19	≥3	2020 (2022)	Yes	Tramadol, diazepam, and promethazine
P-20	≥3	2021 (2022)	No	Naproxen and clarithromycin

^a^ED: eye drops.

### Participants’ Perception About the Web-Based Availability and Risks of Fake Medicines

All but one of the participants (ie, 19/20, 95% participants) were aware of the presence of fake medicines on the internet and the associated risks. The following quotes show examples of responses given by the participants when they were asked about their knowledge of fake medicines available on the web and the potential danger of these medicines:

You see a lot of them on different websites like oh brain force or actively encourage 60% better brain activity...I think the main risk of them is just you being conned out of your money. That’s also fake medications that can actively lead to problems with your body. Uh, you could be taking a medication that makes you infertile.

P-4, line 396

I think these products are marketed as containing certain ingredients or having some health benefits and they are actually missing those key ingredients...they are available online...they don’t have the right ingredients, or they don’t have them in a high enough dosage or they just contain ingredients which are bad for any person to take.

P-11, line 329

### Factors That Influence Consumers’ Decision to Purchase POMs on the Web

#### Overview

**Figure 3:**
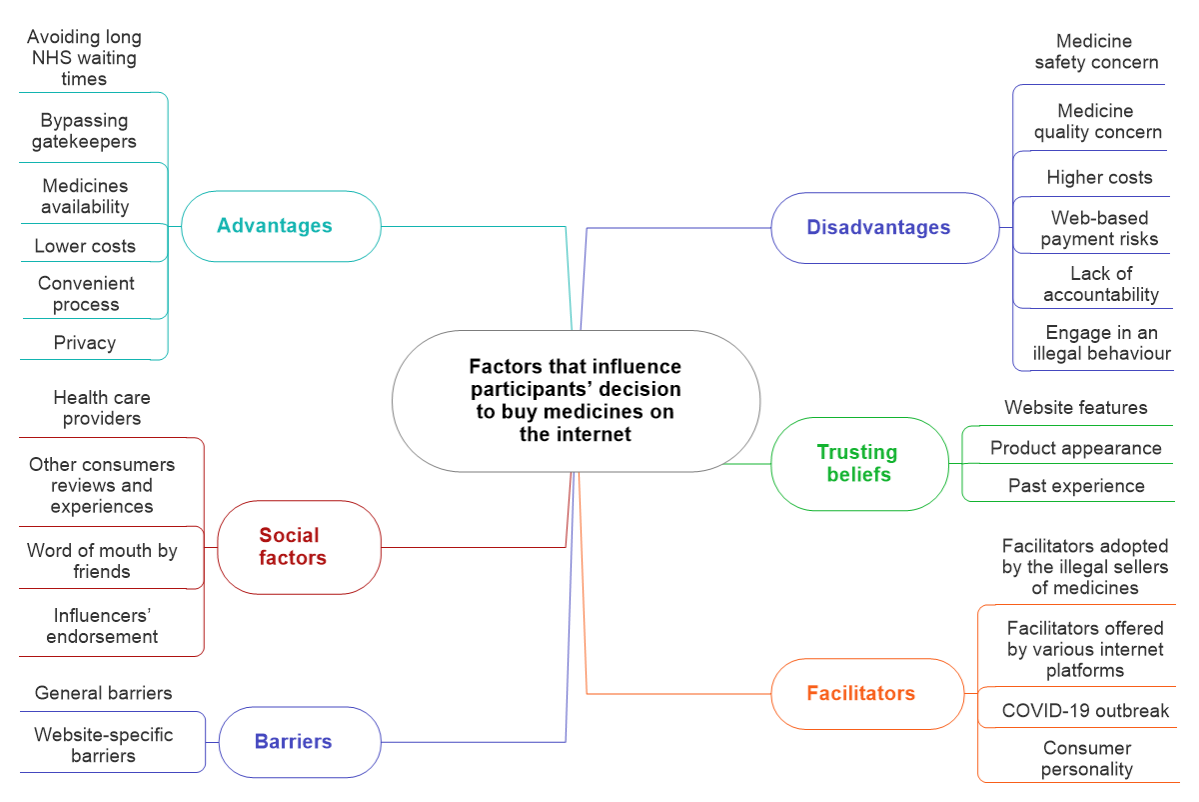
Analysis map. NHS: National Health Service.

**Figure 4:**
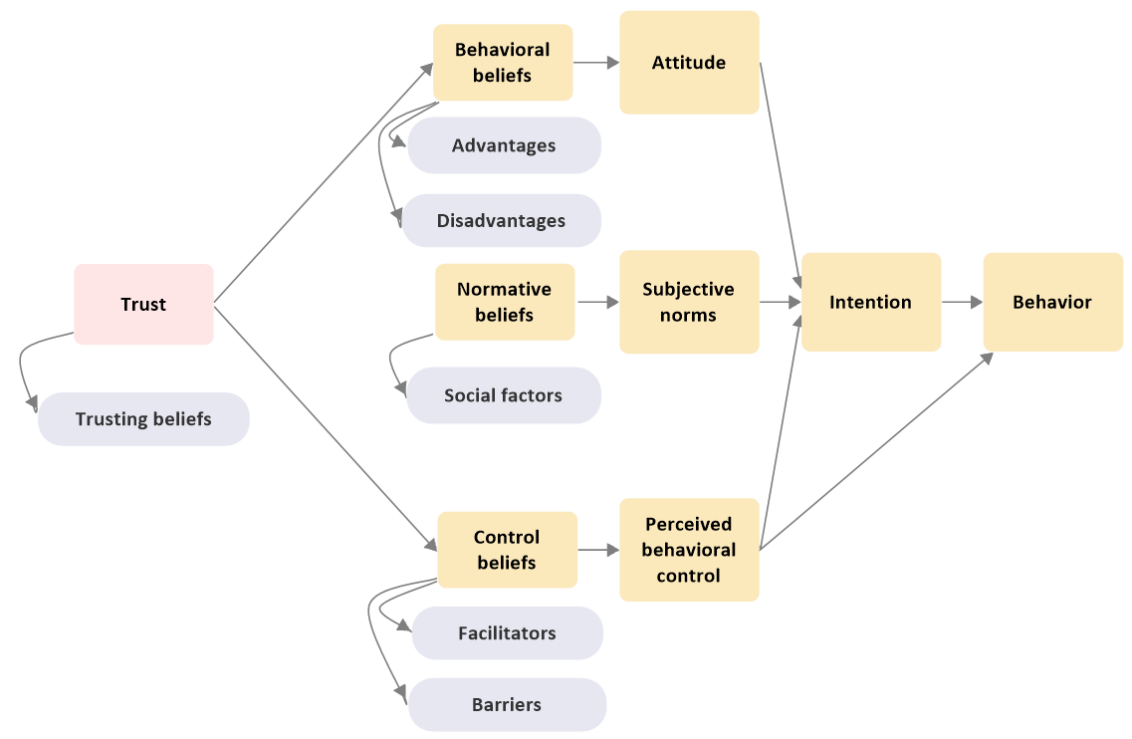
Superordinate themes mapped against the constructs of the theory of planned behavior.

Six superordinate themes were identified from the thematic analysis. These themes represent the range of factors that influence consumers’ decision to make a web-based purchase, without involving the physician, of POMs or medicines that have the potential to be misused or require a higher level of medical oversight before the purchase. The 6 superordinate themes were advantages of purchasing medicines from the internet (advantages), disadvantages of purchasing medicines from the internet (disadvantages), social factors that could influence the decision to make the purchase (social factors), factors that facilitate the web-based purchase (facilitators), factors that prevent or delay the web-based purchase (barriers), and factors that lead consumers to trust in the web-based supplier of medicines (trusting beliefs). Figure 3 illustrates the map resulting from the thematic analysis. All the superordinate themes identified through this analysis could be mapped deductively against the constructs of the extended TPB model, which included the construct of consumers’ trust in web-based suppliers of medicines (trusting beliefs), as shown in Figure 4.

#### Advantages and Disadvantages: Behavioral Beliefs

One of the superordinate themes was the perceived advantages and positive outcomes that consumers might expect if they decide to buy medicines on the web without involving their physician (Table 2). For instance, some of the participants discussed how buying medicines on the web could help them avoid long National Health Services (NHS) waiting times (ie, the time it takes for a patient to receive treatment after being referred for treatment) [36]. The NHS Constitution states that patients should wait no longer than 18 weeks from general practitioner’s referral to treatment. By contrast, if they select the web-based source, they could obtain their medicines within days to weeks. The following example illustrates this point from the perspective of a transgender participant who would otherwise face a long wait for the hormonal medicine they wanted:

I bought Spiro and Estrofem online...You’re asking why I do this...I live in <name of large city>. <name of large city> has its own GIC (Gender Identity Clinic). Its target waiting time is 18 weeks...How long do you think the actual waiting time is in weeks? It's 166 weeks. You have to wait four years to maybe get a referral.

P-2, line 85

Another advantage discussed by some participants was that they could obtain POMs without the need for prescriptions in the first place and thus bypass any gatekeepers, that is, physicians who might refuse to prescribe the POMs, which is illustrated in the following quote:

I looked online and that’s how I came to buy online because they had an awkward GP who wouldn’t give me another prescription for a couple of weeks. So, I went online and bought it and that was the reason.

P-18, line 156

The web-based availability of medicines that are not licensed in the United Kingdom or not obtainable via the NHS for other reasons (eg, out of stock or branded medicines not permitted to be prescribed by brand) motivated some participants to purchase POMs on the web instead of through a safe licensed route, as illustrated by the following quote:

Some medicines from America are not available in the UK for various reason because they are not licensed, I suppose, I don’t know how this works. So, you can get access to medicines which are not normally available on the local market.

P-11, line 148

Some participants frequently discussed the point that obtaining medicines on the web costs less, especially if they otherwise had to pay for their prescriptions either privately or via the NHS prescription charge. The NHS prescription charge is set by the UK government as a levy, and in England, this is equal to £9.35 (US $11.34) per item [37]. The following quote illustrates this point regarding costs:

You can get about like 2 weeks to a month’s worth of estrogen online for £10. It is very very cheap. This is basically what you’d pay in a prescription pharmacy for the same medication.

P-4, line 224

Another advantage that also influenced most participants to buy POMs on the web was that the purchase was convenient and easy, that is, “with just one click,” their medicines were delivered promptly to their homes, as indicated by the following quote:

It’s quick, simple and no need to leave the house, very little forms to fill in, very little intrusion of your privacy, easy to pay.

P-10, line 314

The privacy of the purchase was also an advantage. Consumers could obtain their medicines without the need for face-to-face interactions or stating their illness in cases where the shame of admitting a condition acts as a barrier to seeking care. For example, disclosing sexual dysfunction, addiction to psychoactive medicines, or using slimming pills for weight loss. The following quote illustrates this:

I think the main benefit is not having to see a doctor. You know if you got a male problem, you know if your doctor is only a young female. Probably wouldn’t want to perhaps discuss certain things with a female doctor.

P-10, line 219

The second superordinate theme was the *disadvantages* of the purchase. The participants pointed out concerns regarding purchasing POMs on the web without involving the physician. For instance, some participants highlighted the use of POMs without the physician’s supervision as a risky process, as medicines could be misused or contraindicated. In addition, some participants expressed a concern regarding the safety of medicines purchased on the web, as they could end up receiving and using fake medicines. The following quotes illustrate these:

If you’re not having a doctor’s supervision with these things, you can’t really tell everything is going alright. Let’s say HRT medication. Are you getting a blood test? and then it could be that your liver enzymes are unstable rates, crazy rates that they aren’t supposed to be, and you’re putting yourself at risk of more liver disease. As a result, you’re putting yourself at risk of blood clots.

P-4, line 182

I guess another danger is if people are just pressing the pills themselves instead of sending actual pills, then they could have anything in them. They could be poisoned, or they could be too strong, or they could be anything.

P-5, line 158

With respect to the quality of medicines purchased on the web, most participants were aware that medicines purchased from the internet could be ineffective, stored in poor conditions, or expired, as illustrated by the subsequent quote:

Obviously you might get something that is just like a placebo. You know it’s absolutely no use at all.

P-8, line 140

The participants also had different beliefs regarding the cost of purchasing medicines on the web, mainly based on where they lived. Whereas some said it was cheaper to buy medicines on the web, others stated that buying medicines on the web costs higher. Some participants, specifically those based in Scotland, Wales, and Northern Ireland, attributed this to the fact that they were obtaining medicines through the NHS free of charges. This is because although people in England pay for prescription fees, prescriptions are free of charge in Scotland, Wales, and Northern Ireland. The following quote illustrates this:

In Scotland most of our prescriptions are free, you know so. If I’m buying online, then I’m paying a lot more.

P-7, line 301

In addition, there was a view that there are high shipment costs for medicines purchased on the web, especially if the medicines are purchased from a website based outside the United Kingdom (shipment costs could reach 3 times the cost of the medicine according to some).

Web-based payment risk was highlighted as another drawback associated with purchasing medicines on the web. One of the participants described how she incurred a “*devastating”* financial loss after the website that she purchased medicines from was shut down and the owner of the website disappeared:

I ordered from a website in Russia which was shut down under a basic like they’re rounding up online websites there, so I lost money on about six months’ worth of HRT. You know, that was a pretty devastating for me.

P-2, line 194

Other participants mentioned additional web-based payment risks, including identity thieves who could illegally access personal information (such as credit card details).

The absence of accountability, where no one can be held accountable if any problems happened with the purchase, was also mentioned, as exemplified by the following quote:

No one accountable because you’re not sure who you bought from, you could pay a lot of money for it, and then getting paracetamol or something like this.

P-18, line 228

In addition, some participants talked about the fear of buying POMs (especially controlled medicines) on the web without involving their physician because this kind of purchase is illegal and engaging in such behavior could lead to imprisonment. In this regard, one of the participants said the following:

I guess that’s the fear and the risk that what you’re doing is wrong, and if it’s in medication that’s particularly controlled, then there’s the big risk of you might be gotten, and you know you’ve been found doing something illegal like if trans men were off buying testosterone without a prescription, they could do jail time for that.

P-3, line 220

One of the participants talked about their worry that medicines bought on the web could come from unknown sources or even be stolen medicines:

It could be that they’re a faulty batch that the provider should have disposed of, and they just handed them to their friends. It could be stolen medicines.

P-9, line 273

**Table 2:** Advantages and disadvantages: behavioral beliefs.

Superordinate theme, subtheme and composition					Participants who highlighted the theme
**Superordinate theme 1: perceived advantages of buying POMs^a^ on the web**					
	**Avoiding long NHS^b^ waiting times**				
		Consumers wanted to avoid long NHS waiting times for various reasons (eg, to save time and because they need the medicine urgently, as they cannot tolerate the pain)		2, 4, 5, 10, 11, 14, and 17	
	**Bypassing gatekeepers**				
		Can obtain POMs on the web when physicians or pharmacist refuse to dispense the medicine		1, 3, 4, 5, 6, 7, 8, 9, 11, 15, 18, and 19	
	**Medicines availability**				
		Availability of medicines that are not licensed in the United Kingdom but licensed in other countries		9, 11, and 13	
		Availability of medicines that are not available in the NHS (out of stock)		3, 5, and 6	
		Availability of extra quantities and bulk purchasing		2, 6, 7, 9, 15, and 18	
		Availability of branded medicines (for those who prefer branded medicines)		9 and 14	
	**Lower costs**				
		Costs lower than the NHS prescription charge		1, 2, 4, 6, 8, 10, 16, 17, and 18	
	**Convenient process**				
		Quick purchase and saves the effort of visiting a physician and pharmacy, as the medicine is delivered to the consumer		1, 3, 6, 7, 8, 9, 10, 11, 12, 13, 15, 16, 17, 18, 19, and 20	
	**Privacy**				
		Avoids embarrassment (not necessary to admit to their illness or need)		1, 3, 5, 6, 9, 10, 12, 14, and 19	
**Superordinate theme 2: perceived disadvantages of buying POMs on the web**					
	**Medicine safety concerns**				
		Lack of medical oversight and its consequences (eg, drug-drug interactions and misuse of medicines)		1, 4, 6, 7, 9, 10, 12, 13, 15, and 17	
		Could be fake medicines		1, 2, 4, 5, 9, 11, 12, 14, 18, 19, and 20	
	**Medicine quality concerns**				
		Potential to receive ineffective medicines, expired medicines, or medicine stored in poor storage conditions		3, 4, 5, 6, 7, 8, 9, 10, 11, 13, 15, 16, and 18	
	**Higher costs**				
		More expensive than the medicines obtained through the NHS (especially in Scotland, Wales, and Northern Ireland) or high delivery costs		3, 7, 11, 12, 13, 14, 18, 19, and 20	
	**Web-based payment risks**				
		Financial losses because the medicine is not useful or because of the risk of a financial scam or the risk of identity theft		1, 2, 3, 5, 7, 8, 9, 11, 13, 14, 15, 17, and 18	
	**Lack of accountability**				
		No accountability exists to ensure the safety of the purchased products		6, 7, and 18	
	**Engage in an illegal behavior**				
		Fear of being caught in possession of a controlled drug		3, 6, 7, 8, 13, 18, 19, and 20	
		Ending up buying stolen medicines		9	

^a^POM: prescription-only medicine.

^b^NHS: National Health Services.

#### Social Factors: Normative Beliefs

This superordinate theme represents the participants’ beliefs about the extent to which other people influence their decision to either purchase or not purchase medicines on the web (Table 3). Some participants believed that one of the key actors who (inadvertently) influenced their decision to make web-based purchases of medicines were health care providers (eg, physicians). For instance, this occurred when a physician refused to dispense a specific medicine to the patient that they thought would be useful to them. In addition, some participants discussed how dissatisfaction with their health care providers drove them to buy medicines on the web. The following quote illustrates this point from the perspective of a participant who experienced amenorrhea (stopped periods) after being infected by the SARS-CoV-2 virus. The hospital lost her blood test results, and the physician asked her to wait until the results were found. She waited for months without a response from the physician, so she chose to buy medicines on the web:

When I did my blood test, they lost my blood test results. Then, they told me it takes a long time to find it. Do you know I live in <name of large city>, but I did the test in <name of large city> and <name of hospital> Hospital because there was no appointment available here, the doctor told me wait for your blood test to decide what to do with you. I waited for 2-3 months, and I didn’t get any answer from the doctor. So, I was feeling very bad, and do you know I searched online for medicine what I can do to help to start my period and they suggested me the progesterone.

Positive or negative customer reviews on different forums also influenced some participants to buy medicines on the web, as illustrated by the subsequent quote:

There are probably review sites that you can refer to. So, there’s a review on the website that could help in everyone or there could be another review site that you could be used that are not attached to the website. But people say, yeah, this is good, this is bad.

P-8, line 101

Some participants’ purchasing decisions were also influenced by a trusted friend who had a positive past purchase experience, as exemplified by the following quotes:

It was actually a friend that had already done it. And, uh, you know advise me to do it because like I said I was in so much pain...Umm so yeah, my friend suggested it and then I researched on Google and uh. Yeah, I decided to do that.

P-19, line 166

Other participants discussed how influencers and public figures who have a large number of followers could influence their decision to turn to the internet to buy a POM (which they would otherwise be unable to obtain from a physician) by shaping their perceptions and prompting them to buy impulsively:

It could be that you get some politicians like Donald Trump saying that hydroxychloroquine is a very good thing, and you get social media celebrities or politicians telling you it’s good to buy them and then it’s not good. So, you might be tricked if you buy them.

P-9, line 258

P-16, line 89

**Table 3:** Superordinate theme 3: social factors—normative beliefs.

Subtheme	Composition	Participants who highlighted the theme
Health care providers	If the physician refused to dispense a specific medicine to the patient. Patient dissatisfaction with the health care provider	1, 2, 5, 6, 7, 9, 16, 18, and 20
Other consumers reviews and experiences	Other consumer reviews shown on the medicine supplier websites	1, 8, 10, 11, 14, 17, and 18
Word of mouth by friends	Advice from a friend who purchased the medicine on the web	4, 6, and 19
Influencers’ endorsement	Medicines endorsed by influencers and public figures	9 and 11

#### Facilitators and Barriers: Control Beliefs

Another superordinate theme was participants’ beliefs about the factors that facilitate the process of purchasing medicines on the web. Several *facilitators* were identified across the participants (Table 4). Some of these facilitators were features of the websites where medicines were sold; for example, web-based accessibility of POMs without the need for a prescription was one of these facilitators for some participants:

So, I think the advantages are the access issues that you can get it. Yeah, and there that you can get it without a prescription.

P-3, line 172

Another facilitator offered by the web-based sellers was the availability of a wide range of medicines, including branded medicines, medicines that are not licensed in the United Kingdom, and medicines that are not available on the NHS. The following quote illustrates this:

Some medicines from America are not available in the UK for various reason because they are not licensed, I suppose, I don’t know how this works. So, you can get access to medicines which are not normally available on the local market.

P-11, line 148

Another facilitator was that the web-based sellers provided multiple payment methods and thus made the checkout easy, as the buyers can select the payment gateway that best suits them. In addition, the participants discussed how the payment process was easy and secure as they could choose to pay using different web-based payment systems that offer refund and purchase protection if the consumers were to come across scammers (enabling consumers to receive partial or full reimbursement, including any shipping costs), as indicated by the following quote:

With PayPal, you don’t have to worry about paying to like a foreign account or anything, because it’s easy to get refunds as well with PayPal, so that’s why it’s easy. It’s so easy to get refund.

P-17, line 334

Other facilitators highlighted by the participants as offered by the web-based sellers included marketing tactics adopted by internet pharmacies such as paid advertising (eg, pop-up advertisements) and direct links that can connect the consumer directly to the pharmacy website in one click. The following quote illustrates this:

There are a lot of pop-up ads that appear, you can find these on Google just with like a simple search isn’t even kind of hard to find medicines.

P-4, line 90

Customer support services offered by the medicine seller’s website were mentioned by one of the participants as another facilitator of web-based purchasing of POMs in relation to medicines with complex use instructions:

There are a customer support people who can help you if your delivery is late or something or if you if you want to ask how I take this.

P-17, line 416

Internet platforms also played a more general role as a facilitator of the web-based purchase of medicines. Some participants discussed how *online support communities* available on different internet platforms facilitate the web-based purchase of medicines by offering advice to others on how to purchase POMs on the web or how to use medicines that require complex instructions of use, as illustrated by the following quote:

There’s a very kind of strong community that exists even within the UK, where people will help people who are wanting to obtain these medications. Obtain them in the safest way possible. It actually benefited me quite a bit because I was looking at how to obtain these medications, and it was platforms, like Twitter and Reddit that actually had groups there who gave me full comprehensive guides on how to obtain these medications.

P-4, line 370

Web-based support communities are internet-based social spaces, such as social media groups or forums, where people come together to obtain and provide information or support [38].

The participants also mentioned different search engines (Google, Yahoo, etc) that helped them find internet pharmacies that offer POMs without the need for prescriptions. Finally, the participants mentioned social media platforms, which allow for easy communication between consumers and the illegal web-based sellers of medicines:

When you scroll through a sort of social media platforms, you see things, I mean we all see it pop ups come, adverts come up and I guess we’re conditioned now not click on the links and to come off it and to go on and go into the website directly

P-14, line 292

The outbreak of the COVID-19 pandemic was also mentioned by some participants as a facilitating condition for the purchase of medicines on the web. Anxiety and fear resulting from the thought of getting infected by the SARS-CoV-2 virus triggered one of the participants to make a panic web-based purchase of hydroxychloroquine:

It was at the start of the COVID...I was really scared, and I was reading all this thing, all this stuff online about how to protect yourself and everything like that. I have bought hydroxychloroquine; I have bought it online. Believe it or not, I’ve not opened it. Believe it or not, but this was bought out of a fear to do the Covid.

P-7, line 90

Other factors included restrictions on people’s movements owing to lockdowns or self-isolation, a sense that medical staff were overburdened during the COVID-19 outbreak, and the spread of misinformation on social media. These factors were discussed by some participants as leading to the web-based purchase of medicines.

A small number of participants discussed their opinions about the personal attributes of consumers who decide to purchase medicines on the web, which they related to risk taking:

My personality type doesn’t really like to follow rules. I know it’s not good to be that way, but I’m more the type of person that I just like to make my own decisions like making informed choice rather than being formed what to do, or watch what somebody else is doing. I’m an independent free thinker and I’m more of a person that will take risks more easily.

P-6, line 638

By contrast, the participants also pointed out several barriers that they have encountered during the purchase. These barriers could either prevent people from buying medicines from a specific website (website-specific barriers) or from the internet in general regardless of which website they select (general barriers). Several website-specific barriers were discussed by some participants; for example, the absence of suitable payment options on a specific website could prevent people from making the purchase. Although some described payment and checkout as an easy and secure process, others stated that the absence of suitable payment options on a website would hinder the purchase. The following quote illustrates this:

A lot of the websites only take payment in Bitcoin and a lot of people don’t really understand Bitcoin, so that’s that kind of makes it a bit more complicated.

P-5, line 119

Another website-specific barrier was the language barrier, which was mentioned by one of the participants in the context of web-based purchase from a non-English website:

Sometimes there’s language barrier because some they’re like Chinese websites or like Russian or like some other websites which have different language.

P-17, line 369

Another barrier highlighted by some participants was that some websites do not have the option of delivering medicines to the United Kingdom:

Sometimes some websites do not deliver to certain countries. I don’t know why. They have lists or lists of countries which they don’t deliver to, so you think you found your medicine and try to buy it. And then when you put your address in, you get the message. Sorry we can’t deliver to that country.

P-11, line 294

In addition, customs and police were mentioned by some participants, as they can hinder supply by closing the websites of unlicensed sellers of medicines. Purchasers would then need to search for a suitable alternative internet pharmacy, which might be difficult to find:

The website that I mentioned first <name removed> which is the one that I used; it was shut down. It was brought up in a newspaper in one of those anti-trans articles, a week or two it had been shut down and gone, and that happened a couple of times with similar websites for that.

P-3, line 369

General barriers could restrain consumers from purchasing medicines on the web in general regardless of which website they select; for example, the complexity of medication instructions was pointed out by some participants as barriers that could prevent the purchase. Despite some participants’ view that web-based support communities provide information on how to use medicines with complex instructions, other participants felt that the prospect of using some medicines or some formulations without the input of professional health care provider was a barrier to them purchasing medicines on the web because they would not know how to use the products without this input (eg, inhalers or injections). Financial capability was also mentioned by a few participants as a barrier that could prevent the purchase of medicines on the web. The following quotes exemplified these points:

The nature of the medication. If it’s a pill, it would merely easier to buy online than buying something that involved injecting like needles. Because you also need to buy the equipment and know how to do it.

P-3, line 376

Money has an influence here; I mean having the money to do it. I know quite a few friends who would have loved to be able to do this and buy medication online for themselves, but they did not have the funds to do it.

P-3, line 373

**Table 4:** Facilitators and barriers: control beliefs.

Superordinate theme, subtheme and composition			Participants who highlighted the theme
**Superordinate theme 4: facilitators**			
	**Facilitators adopted by the illegal sellers of medicines**		
		POMs^a^ accessibility (accessibility to POMs without the need for a prescription)	3, 4, 7, 9, and 18
		Medicine availability (provide a wide range of medicines, including branded medicines, medicines that are not licensed in the United Kingdom, and medicines that are not available in the NHS^b^)	5, 6, 9, 11, 13, and 14
		Provision of a variety of easy and secure payment options as well as the availability of web-based payment systems that offer refund and protection (eg, PayPal’s Purchase Protection)	6, 7, 9, 17, 20, 8, and 17
		Customer support offered by the illegal sellers’ websites	17
		Illegal sellers’ marketing (pop-up advertisements and direct links)	4, 11, and 13
		Ability to make bulk purchasing without limits.	2, 6, 7, 9, 15, and 18
	**Facilitators offered by various internet platforms**		
		Social media platforms (easy communication)	5, 9, 11, and 14
		Review websites	1, 8, 10, 11, 14, 17, and 18
		Search engines (eg, Google), which facilitate the searching process	4 and 9
		Signposting by support communities available on different internet platforms	4 and 5
	**COVID-19 outbreak (facilitating conditions)**		
		Anxiety and fear due to COVID-19	7
		Mobility restrictions owing to lockdown or self-isolation	5, 14, 18, and 20
		Overburdened medical staff during the COVID-19 outbreak	4, 9, 10, 14, 16, 17, 18, and 20
		Spread of misinformation on social media and other platforms	7 and 9
	**Consumer personality**		
		Willingness to take risk	6 and 19
**Superordinate theme 5: barriers**			
	**General barriers**		
		Complexity of medication instructions: POMs that require complex instructions for use (eg, injections)	3 and 18
		Financial capabilities: cannot afford to pay for medicines available on the web	3 and 4
	**Website-specific barriers**		
		Absence of suitable payment options	5, 9, 11, 12, 13, and 17
		Language barrier if the website is in a non-English language	17
		Delivery problems such as the websites that do not deliver to the United Kingdom	5, 11, and 19
		Illegal website closures or seizure of the parcels at the UK borders by organizations stopping the purchase of POMs on the web (eg, customs and the Interpol)	2, 3, 5, 7, and 11

^a^POM: prescription-only medicine.

^b^NHS: National Health Services.

#### Trusting Beliefs

Several factors that increase peoples’ *trust* in internet pharmacies were also highlighted by the participants (Table 5). Half of the participants discussed how specific website features led them to trust internet pharmacies. These include the availability of detailed and accurate product information on the website. Website appearance (eg, high-quality photography, language used, and spelling accuracy) was also discussed by some participants as a factor that increases their trust level. In addition, the presence of contact information on the seller’s website, such as address and phone number, was highlighted as a factor increasing consumers’ trust in the web-based sellers of medicines. Finally, websites that collect a thorough medical history of consumers before the purchase were believed to be more trustworthy. The following quotes exemplify this theme (ie, website features):

I think what made trust the website was that it looked professional. It didn’t look like, for example, there weren’t any spelling errors.

P-14, line 637

I prefer sites that do actually have an address and also a telephone number. If you want to make contact, you know there’s a number

P-18, line 139

There is enough information about the medicine, the way they work, maybe some reviews from clients, then, I think they tend to look up genuine and trustworthy.

P-11, line 69

Other participants thought that if the medicine obtained looked identical or similar to the drug they wanted to buy, this influenced their trust in the purchase:

It was quite a long process because I didn’t just go to a site and just buy it. I was careful, I mean, you probably shouldn’t buy prescription medicines online, but I was very careful. Yes. And when it arrived, it looked just the same packet as the one I would have got from my GP.

P-18, line 184

Positive previous purchase experience was also indicated by some participants as a factor that increased their trust in the internet pharmacies. Some participants discussed how they trusted websites from which they have had a safe and secure previous purchase:

I think it’s habit forming, if you have a positive experience with that, you’re more likely to repeat that experience. And every time you get a positive experience, you’re just gonna back yourself or back your own thinking up on it and go. It’s absolutely fine. It’ll be totally OK. Obviously, it’s almost that way of like. I think it’s dependent on personal experience.

P-20, line 465

Other participants trusted websites suggested to them by trusted friends or judged whether a website was trustworthy by relying on other customers’ reviews. The following quotes illustrate these points:

No, it was actually a friend that had already done it. And, uh, you know advise me to do it because like I said I was in so much pain.

P-19, line 166

I think customer reviews or so seem to play a really large part in the honesty and integrity of a company selling goods online.

P-14, line 114

**Table 5:** Superordinate theme 6: factors that affect consumers’ trust in the web-based seller—trusting beliefs.

Subtheme	Composition	Participants who highlighted the theme
Website features	Product information availability and accuracy	11 and 18
	Appearance	3, 5, 6, 14, and 18
	Display of the seller’s contact information	1 and 18
	Collection of medical history before purchase	3, 12, 13, 15, and 18
Product appearance	Medicines offered by the internet pharmacy look similar to genuine medicine	11, 16, 18, and 20
Positive previous purchase experience	Had a safe and secure previous purchasing experience	6, 18, and 20
	Word of mouth from friends (recommendation by a friend who had purchased medicines from the website)	4, 6, and 19
	Other customers’ positive experiences (positive customer reviews)	1, 8, 11, 14, 17, 18, and 20

## Discussion

### Principal Findings

This in-depth, theory-based, qualitative study of consumers’ actual experiences identified a breadth of factors that influence people’s decision to purchase POMs on the web. This in-depth, theory-based qualitative study of consumers’ actual experiences identified a breadth of factors that influence people’s decision to purchase POMs on the web. Participants in this study purchased various types of POMs using the internet despite their understanding of the potential risks associated with this kind of purchase, including falling prey to receiving fake medicines. The 6 major themes identified covered the perceived advantages and disadvantages of purchasing medicines on the web, social influencing factors, facilitators, barriers, and factors that lead consumers to trust the web-based seller.

One unanticipated key finding of this study, as mentioned earlier, was that the participants did seem to know about the potential risks associated with purchasing POMs on the web, including awareness of the widespread availability of fake medicines on the internet, yet they made the purchase from unregulated web-based markets and put themselves at risk of buying fake medicines. This finding is contrary to a previous study that suggested that people are purchasing medicines on the web because they were unaware of the risk of the purchase [39]. Therefore, there are other factors that influence UK consumers to either buy or not to buy medicines on the web. One of these factors was the advantages of the purchase. These UK-based participants were driven by what they perceived as positive outcomes of the web-based purchase, such as convenience and medicine availability. These 2 advantages are in line with the findings of several other research studies conducted in different countries, including the Middle East, Europe, the United Kingdom, and the United States [1,10,16,17,40-42]. An interesting finding is that in some situations, the long NHS waiting time (ie, long time for patients to be treated) could drive people to end up buying prescription medicines on the web, including potentially fake medicines. Some participants believed that avoiding the long NHS waiting time was a potential advantage of the web-based purchase. Therefore, those participants were driven to access an unregulated web-based market (a potential source of fake medicines) to self-medicate and receive their purchase expediently.

Another novel finding of this study is the influence of cost on consumers’ decisions to purchase POMs on the web. The beliefs of some of this UK-based study’s participants regarding the costs of web-based purchase were contradictory to previous non-UK studies reporting that people might be motivated to purchase on the web because it is cheaper [1,14,18,43]. Some considered purchasing medicines on the web to come at a lower cost, especially if the price was cheaper than the NHS prescription charge. For others who were exempt from prescription charges, purchasing medicine on the web was not preferable because of the higher cost compared with obtaining their medicines for free through the NHS.

Although the participants in this study highlighted the advantages of purchasing POMs on the web and how these advantages influenced their decision to make the purchase, they also discussed how purchasing POMs on the web was associated with many disadvantages, as the efficacy and safety of medicines cannot be guaranteed if they are purchased on the web. They also mentioned payment risks and that if any unpleasant consequences occurred (eg, unexpected side effects, failure in delivery, or a financial scam), no one could be held accountable, except for the consumers themselves. Therefore, in the United Kingdom, policy makers could address this absence of accountability by holding the internet search engines and social media platforms responsible if they permit illegal web-based sales of medicines to happen through them. Therefore, this study supports what Liang and Mackey [43] suggest about the importance of addressing accountability to ensure the safety of the people who look to purchase medicines on the web, which also reduces the chance of fake medicines being purchased.

Another important finding was that health care providers (eg, physicians and pharmacists) can (inadvertently) lead people to purchase medicines, and thus potentially fake medicines, from the internet. For example, if a physician refuses to prescribe a specific medicine to a patient that the patient thinks would be useful to them, then the patient might start searching for alternative web-based sources, whose safety cannot be assured. This finding is in line with a study conducted in the United States that found that people who use tramadol were motivated to use unregulated web-based markets of medicines as a source of tramadol when they could not find a physician who would prescribe it [18]. However, health care providers could also play a beneficial role. In a Maltese study, health care providers played a crucial role in determining the source of medicines purchased by consumers, which could decrease the possibility of purchasing medicines from unregulated web-based sources [15]. In addition, pharmacists can play a crucial role in preventing consumers from purchasing medicines on the web by increasing their awareness of the dangers of this type of activity [1].

Various facilitators of and barriers to the process of purchasing POMs from the internet were also identified in this study. Although consumers could encounter many barriers that might delay or prevent the web-based purchase, some facilitators offered by the web-based sellers of medicines or the different internet platforms could actually assist consumers in overcoming these barriers. For example, some people believed that web-based payment comes with many risks and is considered difficult in the absence of suitable payment methods; some websites, for example, only accept payment with Bitcoin, which many people are unfamiliar with. However, some web-based sellers have helped consumers overcome this by offering an easy and secure payment option via their website, meaning that a suitable payment gateway will be there waiting for them. In addition, web-based payment systems that offer refunds and protection in case of a financial scam (eg, PayPal [PayPal Holdings, Inc]) could facilitate the process by reassuring consumers that compensation is available for any financial losses incurred. Another example is the facilitating role played by web-based support communities that are accessible via different internet platforms. These support communities help consumers who do not know how to use complex medicines, especially in the absence of medical oversight, to overcome this barrier by providing them with information on how to use them. There is a shortage of literature exploring the key issues identified in this study.

The web-based accessibility of POMs without the need for prescriptions was one of the facilitators that was frequently discussed by participants in this study. What is surprising is the wide range of POMs available and accessible on the web to UK consumers, including hormone replacement therapy, antibiotics, or even high-risk controlled medicines, which in the United Kingdom normally require additional checks before being dispensed in a brick-and-mortar pharmacy. This finding broadly supports the work of other studies [4,5], which also indicate that a range of POMs are available on the web to UK consumers without the need for a prescription and without medical supervision. This is despite the fact that purchasing such medicines on the web and consuming them in the absence of medical supervision can expose consumers to many serious risks, such as medicine abuse, medicine misuse, and the possibility of receiving fake medicines that are ineffective or harmful.

An interview study conducted in Nigeria found that social media platforms, including Facebook, Twitter, Instagram, and WhatsApp, were used by the illegal web-based sellers of medicines as a place to easily sell and market their products [44]. This study’s findings are in line with this, as the participants reported that social media platforms facilitated their purchasing of POMs on the web by providing an easy and direct communication channel between them and the illegal sellers of medicines. Similarly, an international study that explored the factors that motivate pregnant women to purchase medicines on the web found that a Facebook group page influenced pregnant women’s intentions to purchase medicines on the web by recommending the purchase [16]. The findings of this study support this notion, as web-based support groups found on the internet (including on social media platforms) were said to facilitate the web-based purchase of medicines by helping consumers use medicines with complex instructions.

During the outbreak of the COVID-19 pandemic, misinformation about COVID-19 cures and medications spread globally, which was described as an “infodemic,” causing desperation and panic among many people, which, in turn, created an opportunity for illegal web-based sellers to produce and sell fake medicines [45,46]. This study is in line with what has been reported about this, as we found that the spread of misinformation on social media during the outbreak of COVID-19 pandemic as well as the anxiety and fear resulting from the thought of getting infected by the SARS-CoV-2 virus were triggers for the participants to purchase medicines on the web.

Trust was found to play a crucial role in shaping consumers’ attitude toward the web-based sellers of medicines. In such a dubious environment, the consequences of trusting the illegal web-based sellers of medicines include the possibility of ending up with fake medicines that might be ineffective or, worse still, toxic or even life-threatening when consumed. Some participants trusted the websites they had previously purchased from if they did not encounter any problems regarding the safety and quality of the medicines purchased. This finding was also reported by Koenraadt and Van de Ven [40]. Others judged whether the medicines purchased were genuine based on the appearance of the medicines and packaging; if the medicine looked similar to a previous genuine medicine that they had obtained, then it *was* genuine as far as they were concerned. In fact, medicines are not a commodity but a highly controlled and manufactured complex substance that requires specific storage conditions and whose integrity, safety, and effectiveness cannot be guaranteed if purchased from an illegal web-based seller [47].

### Strengths, Limitations, and Future Research

The strength of this study is that it is the first in-depth qualitative study that focuses on consumers’ actual experiences (ie, experiences of purchasing medicines on the web without input from a qualified health care professional) and explores their behaviors by highlighting the complexity of personal beliefs and motivations toward buying POMs or other high-risk medicines on the web in the United Kingdom. It also explores perceptions about the web-based availability and risks of fake medicines, which could guide authorities fighting the web-based trading of fake medicines to increase the efficacy of their public awareness campaigns.

A limitation of this study is that the participants may have felt reluctant to fully disclose their web-based medicine-buying behavior if it was illegal or to talk openly about buying medicines on the web without consulting a prescriber, based on the notion that it might be judged harshly by the researcher. Another limitation is that although the interviews provided detailed answers to the study questions because the they were continued until data saturation was reached, the findings might not necessarily be generalizable, as the study was conducted on a small sample. However, the theory used in this study (ie, the TPB) has well-established guidelines for developing a questionnaire, allowing the factors identified in a qualitative study such as the current one to be formally verified and generalized at a later date [22].

### Conclusions

Despite the efforts made by public awareness campaigns in the United Kingdom to educate consumers on how to purchase medicines from the internet safely, people in the United Kingdom are still purchasing POMs on the web without input from a qualified health care professional, although they are aware of the risks associated with this activity, including the risk of purchasing fake medicines.

This study provides an in-depth understanding of the reasons why people take risks and buy POMs from web-based sellers, who are a potential source of fake medicines. Identifying these reasons provides the basis for awareness campaigns that are relevant and fit for purpose. The current findings can be used to design interventions and behavior change strategies for minimizing and preventing people from buying POMs from the internet, thereby preventing the purchase of fake medicines. The next step is to develop a questionnaire based on the themes generated in this study to verify and generalize the findings by collecting views about this phenomenon from a wide and representative sample of participants in the United Kingdom.

## Appendix

Multimedia Appendix 1 The Standards for Reporting Qualitative Research.

Multimedia Appendix 2 Recruitment poster.

Multimedia Appendix 3 The web-based recruitment screener.

Multimedia Appendix 4 Interview schedule.

## Abbreviations

NHS: National Health Services
POM: prescription-only medicine
TPB: theory of planned behavior

## Data Availability

Data are available upon request.
