# Supplementary material for: Reasons That Lead People to End Up Buying Fake Medicines on the Internet: Qualitative Interview Study
Source: JMIR Form Res. 2023 Feb 16;7:e42887. doi: 10.2196/42887 (PMC9982721; doi:10.2196/42887)
Supplement: Multimedia Appendix 2 [file formative_v7i1e42887_app2.pdf]

# Multimedia appendix 2

## PARTICIPANTS NEEDED FOR ONLINE INTERVIEWS

University of Reading

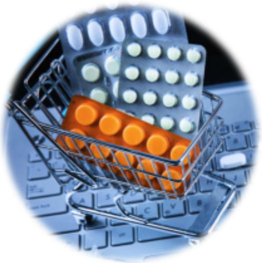

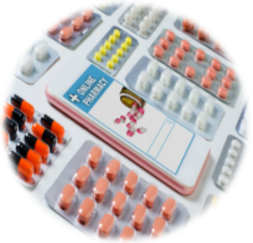


### HAVE YOU EVER BOUGHT A PRESCRIPTION MEDICINE ONLINE?

Have you ever bought any prescription medicine online without involving your doctor?

If so, please come and join my study!

More and more people are turning to the internet to meet their medicine needs. This comes with advantages and disadvantaged. I am interested in hearing your views on this topic and learning about your own experiences.

### QUALIFIED PARTICIPANTS MUST

- Be over 18 years old.
- Be based in the UK.
- had bought any prescription medicine using an online pharmacies, without a prescription.

### WHERE?

The interview will be conducted online via *Microsoft Teams* and will last for up to 1 hour.

You will be reimbursed for reasonable expenses (£20).

### ARE YOU INTERESTED?

Please contact me and I will send you further information.
Hamzeh Almomani
h.q.m.almomani@pgr.reading.ac.uk

***Supervisors***

Prof. Parastou Donyai
p.donyai@reading.ac.uk

Dr. Nilesh Patel
nilesh.patel@reading.ac.uk
