# Supplementary material for: Reasons That Lead People to End Up Buying Fake Medicines on the Internet: Qualitative Interview Study
Source: JMIR Form Res. 2023 Feb 16;7:e42887. doi: 10.2196/42887 (PMC9982721; doi:10.2196/42887)
Supplement: Multimedia Appendix 3 [file formative_v7i1e42887_app3.pdf]

Multimedia appendix 3

The online recruitment screener questions:

1. **How old are you?**

   Under 18 (screen out, ***i.e., excluded from the study***)
   18-29
   30-39
   40-49
   50-59
   60-69
   >=70

2. **Where in the UK do you live?**

   (England/Scotland/Wales/NI)

3. **What is your gender?**

   (Male, Female, Others, Prefer not to say)

4. **What is your ethnicity?**

   (White British, Asian British, Black British, Others)

5. **Have you ever bought a prescription medicine from the internet without involving the doctor?**

   Yes
   No (screen out)

6. **What is the name(s) of the prescription medicines you bought online? Please write down all you have bought**

   1.
   2.
   3.
   4.
   5.

7. **How many times have you bought prescription medicines online?**

   Once
   Twice
   Three times or more

8. **Where were you living when you bought the prescription medicines online?**

   In the UK
   Outside of the UK (Screen out)

9. **When was the last time you bought prescription medicines online?**

This year (2022)
2021
2020
2019
2018 or before (Screen out)

10. **Have you ever faced any problems with the prescription medicines you bought online?**

    Yes
    No

11. **Thank you for participating so far! You have qualified to participate in the interview!**

    I am looking to recruit a range of people for my interview study with people who have bought prescription medicines from the internet. My aim is to explore what motivates people to buy medicines from unlicensed online pharmacies, and people's awareness about the dangers of fake medicines that could be available online. A detailed Participant Information Sheet and consent form will be sent before any interviews.
    The interview would take up to 1 hour and the incentive for this would be £20.00 paid into your Panelbase account 1-2 days after the interview.
    Would you be happy to proceed?

    Yes
    No (screen out)

12. **Question about participants availability. (Date and time)**

13. **Thank you for your time, please enter the following details and the University of Reading will be in touch with an information sheet as well as a consent form that you will need to sign and email back. Your details will only be used to contact you about the interview and will not be used for any other purposes.**

    Name:
    Email:
    Mobile number:

    It you are not interested anymore please click here. (Screen out)

14. **Great! You will be contacted by the University of Reading shortly, to arrange the interview date and time! Please click the button below to submit your details.**

    Submit
