# Supplementary material for: Reasons That Lead People to End Up Buying Fake Medicines on the Internet: Qualitative Interview Study
Source: JMIR Form Res. 2023 Feb 16;7:e42887. doi: 10.2196/42887 (PMC9982721; doi:10.2196/42887)
Supplement: Multimedia Appendix 4 [file formative_v7i1e42887_app4.pdf]

Multimedia Appendix 4

Interview Schedule

Introduction

Please note that:

- This interview will be recorded for scientific research purposes.
- All the information collected during this interview will be treated confidentially.
- There are no right or wrong responses; we are just interested in your opinion.

Before we start, do you have any questions?

This study will explore people views about buying prescription medicines online without involving the doctor. In other words, we want to explore what drive people to do this, or what are the reasons that lead them to do this.

Question 1: What do you know about the rules that control buying of medicines? what are the different types of medicines based on the level of control?

Question 2: What type of medicines could be available to buy online?

Question 3: What sort of websites do you think would offer medicines online?

Question 4: Do you think all websites that offer Prescription Only Medicines have the licensed to sell those medicines online? Could you explain your thoughts on this please?

Question 5: How do you think people could obtain the Prescribed-Only Medicines online?

Question 6: How do you think people might recognise if the website they decide to buy Prescribed only medicines from is licenced or not?

Question 7: As you indicated your interest to take a part in this study, I assume that you have bought any Prescribe Only Medicines (POMs) online but without involving the doctor (from unlicensed websites). what was that medicine(s), and what was that experience and how did you buy that medicine?

Question 8: What made you purchase a prescription medicine online without involving the doctor? Is it only you who decide to buy POMs online? Does anyone else influence you to do this ? or are there any other factor made do this?

Behavioural Beliefs (Advantages and disadvantages of the purchase)

Question 9: What do you think are the disadvantages or the risks of buying medicines from unlicenced websites?

Question 10: What do you think are the advantages or the benefits that people could get if they decide to buy a Prescription Medicine online from unlicenced websites?

Normative Beliefs (Social factors)

Question 11: Which individuals or groups of people do you think would encourage the purchasing of Prescription Medicines online from unlicenced websites? (Why do you think they may do this?)

Question 12: Which individuals or groups might discourage the purchasing of Prescription Medicines online from unlicenced websites? (Why do you think they may do this?)

Question 13: If someone that you care about decides to buy a prescribed medicines online from unlicenced websites, will you agree with this or will you stop them from doing this? Why?

Control beliefs (Facilitators and barriers)

Question 14: Do you think buying medicines from unlicenced websites is easy? What are the things that make it easy process? What are the things or circumstances that facilitate or encourage people to buy prescription medicines from unlicensed websites?

Question 15: what are the things make it difficult or what are the barriers of buying prescription medicines online?

Why have they trusted the online suppliers?

Question 16: Based on your purchasing experience, what are reasons that made you trust the online supplier that you have purchased medicines from? What are the characteristics and features of that online suppliers that made you trust them?

Question 17: What do you understand when I use the words 'fake medicines? Do you think fake medicines could be available online? What do you think about the risks of this?

Question 18: Some people might be aware about the availability of fake medicines online, however, they are still buying their prescription online. What do you the reasons that make do this?

Debriefing

The title of this study is "Why do people end up buying fake medicine online?" I want to thank you for taking your time in participating in this study

The study results will be used in my Ph.D. thesis and may be presented at conferences or published in an academic journal. Please note that you are free to withdraw from this study up to one month after the interview; this is because after one month, your opinions will be combined with other participants opinions, and it will be impossible to exclude yours specifically.

Please note that all your personal information will remain confidential and will never be shared with any other party. Your interview will only be accessible to me and my supervisors.

The aim of this study is to identify the factors that motivate people to buy medicines online which could lead them, inadvertently, to buy fake medicines online. Exploring these factors will help in changing/controlling the purchasing of fake medicines online.
